# Identification of Four Mouse Diabetes Candidate Genes Altering β-Cell Proliferation

**DOI:** 10.1371/journal.pgen.1005506

**Published:** 2015-09-08

**Authors:** Oliver Kluth, Daniela Matzke, Anne Kamitz, Markus Jähnert, Heike Vogel, Stephan Scherneck, Matthias Schulze, Harald Staiger, Fausto Machicao, Hans-Ulrich Häring, Hans-Georg Joost, Annette Schürmann

**Affiliations:** 1 Department of Experimental Diabetology, German Institute of Human Nutrition Potsdam-Rehbruecke, Nuthetal, Germany; 2 German Center for Diabetes Research (DZD), Neuherberg, Germany; 3 Institute of Pharmacology and Toxicology, University of Braunschweig, Braunschweig, Germany; 4 Department of Molecular Epidemiology, German Institute of Human Nutrition Potsdam-Rehbruecke, Nuthetal, Germany; 5 Department of Internal Medicine, University Hospital Tübingen, Tübingen, Germany; 6 Institute for Diabetes Research and Metabolic Diseases of the Helmholtz Center Munich at the University of Tübingen, Tübingen, Germany; 7 Department of Pharmacology, German Institute of Human Nutrition Potsdam-Rehbruecke, Nuthetal, Germany; Stanford University School of Medicine, UNITED STATES

## Abstract

Beta-cell apoptosis and failure to induce beta-cell regeneration are hallmarks of type 2-like diabetes in mouse models. Here we show that islets from obese, diabetes-susceptible New Zealand Obese (NZO) mice, in contrast to diabetes-resistant C57BL/6J (B6)-ob/ob mice, do not proliferate in response to an in-vivo glucose challenge but lose their beta-cells. Genome-wide RNAseq based transcriptomics indicated an induction of 22 cell cycle-associated genes in B6-ob/ob islets that did not respond in NZO islets. Of all genes differentially expressed in islets of the two strains, seven mapped to the diabesity QTL *Nob3*, and were hypomorphic in either NZO (*Lefty1*, *Apoa2*, *Pcp4l1*, *Mndal*, *Slamf7*, *Pydc3*) or B6 (*Ifi202b*). Adenoviral overexpression of *Lefty1*, *Apoa2*, and *Pcp4l1* in primary islet cells increased proliferation, whereas overexpression of *Ifi202b* suppressed it. We conclude that the identified genes in synergy with obesity and insulin resistance participate in adaptive islet hyperplasia and prevention from severe diabetes in B6-ob/ob mice.

## Author Summary

Complex genetic determinants contribute to an inherent susceptibility of type 2 diabetes, characterized by insulin resistance, a dysfunction and loss of insulin-producing beta-cells. We compared the islet expression profile and the genome of two obese mouse strains that react differently when receiving a caloric enriched diet. One mouse (B6-ob/ob) is able to compensate by increasing the beta-cell mass, whereas the other (NZO) develops hyperglycemia due to beta-cells loss. Focusing on differentially expressed genes that are located in susceptibility locus for diabetes and obesity on chromosome 1 we found 6 genes to be only expressed in islets of the diabetes-resistant mouse and one to be exclusively present in islets of the diabetes-prone mouse. Among these, the overexpression of 3 genes (*Lefty1*, *Apoa2*, and *Pcp4l1*) increased and that of *Ifi202b* decreased the division of primary islet cells. In summary, our data provide new insights into genes inducing or inhibiting islet size and thereby participate in the pathogenesis of type 2 diabetes.

## Introduction

The hallmark of type 2 diabetes (T2D) is a relative hypoinsulinemia which is unable to compensate insulin resistance and is a result of beta-cell failure [1]. In mouse models of obesity, differences in beta-cell adaptation to increased insulin demand are due to an underlying genetic background causing either diabetes susceptibility or resistance. B6-ob/ob mice lacking leptin on the C57BL/6 background become obese and insulin resistant but do not develop hyperglycemia because of massive beta-cell proliferation and high serum insulin levels. In contrast, the C57BLKS mice carrying the ob/ob mutation do not adapt to increased insulin requirements and die of severe hyperglycemia [2,3].

Here we compared the genetics and pathomechanisms of the diabetes resistant B6-ob/ob mouse with the diabetes-prone New Zealand Obese (NZO) mouse that represents an excellent model for polygenic obesity and type 2 diabetes and resembles the human disease. By positional cloning we have previously identified the adipogenic and diabetogenic genes *Tbc1d1* [4], *Ifi202b* [5], and *Zfp69* [6] of which the human orthologues also appear to be involved in the progression of human obesity and T2D [6–8].

Interestingly, restriction of carbohydrates protects diabetes-prone mice (NZO, db/db on C57BLKS background) from hyperglycemia, but re-exposure to carbohydrates causes rapid development of hyperglycemia and beta-cell apoptosis within a few days [9–11]. We have recently developed a dietary intervention which allowed a very fast and synchronized beta-cell failure in NZO-mice. By feeding a carbohydrate-free diet for about 15 weeks and subsequent treatment with carbohydrates we were able to follow early pathogenic alterations in the islets in a time course of 16 and 32 days. We discovered a rapid induction of hyperglycemia, a decreased AKT signaling and subsequent induction of apoptosis in the islets of Langerhans from NZO mice [10,12].

In the present study we used an integrated approach to identify novel disease genes. We compared the transcriptome profile of islets from NZO and B6-ob/ob mice that were treated with carbohydrates only for two days subsequent to the carbohydrate-free feeding and combined the expression pattern with a MetaCore enrichment pathway analysis and with QTL identified in the F2(NZOxB6) [13]. Several transcripts of cell-cycle regulation are likely to be associated with the pathogenesis of diabetes because their expression in islets of Langerhans is non-responsive to an *in-vivo* glucose challenge in the diabetes-sensitive strain. Mapping of differentially expressed genes to the major diabetes QTL *Nob3* on chromosome 1 identified 15 genes of which 7 exhibited an exclusive expression in one strain. Direct evidence was obtained for four genes of which three appear to be involved in the compensatory capacity of B6-ob/ob islets: *Lefty1*, *Pcp4l1*, and *Apoa2* exclusively expressed in B6-ob/ob islets increased proliferation. In contrast, *Ifi202b* which is not expressed in B6 suppressed proliferation when overexpressed in primary islet cells.

## Results

### Induction of proliferation in islets of diabetes-resistant B6-ob/ob mice

**Fig 1:**
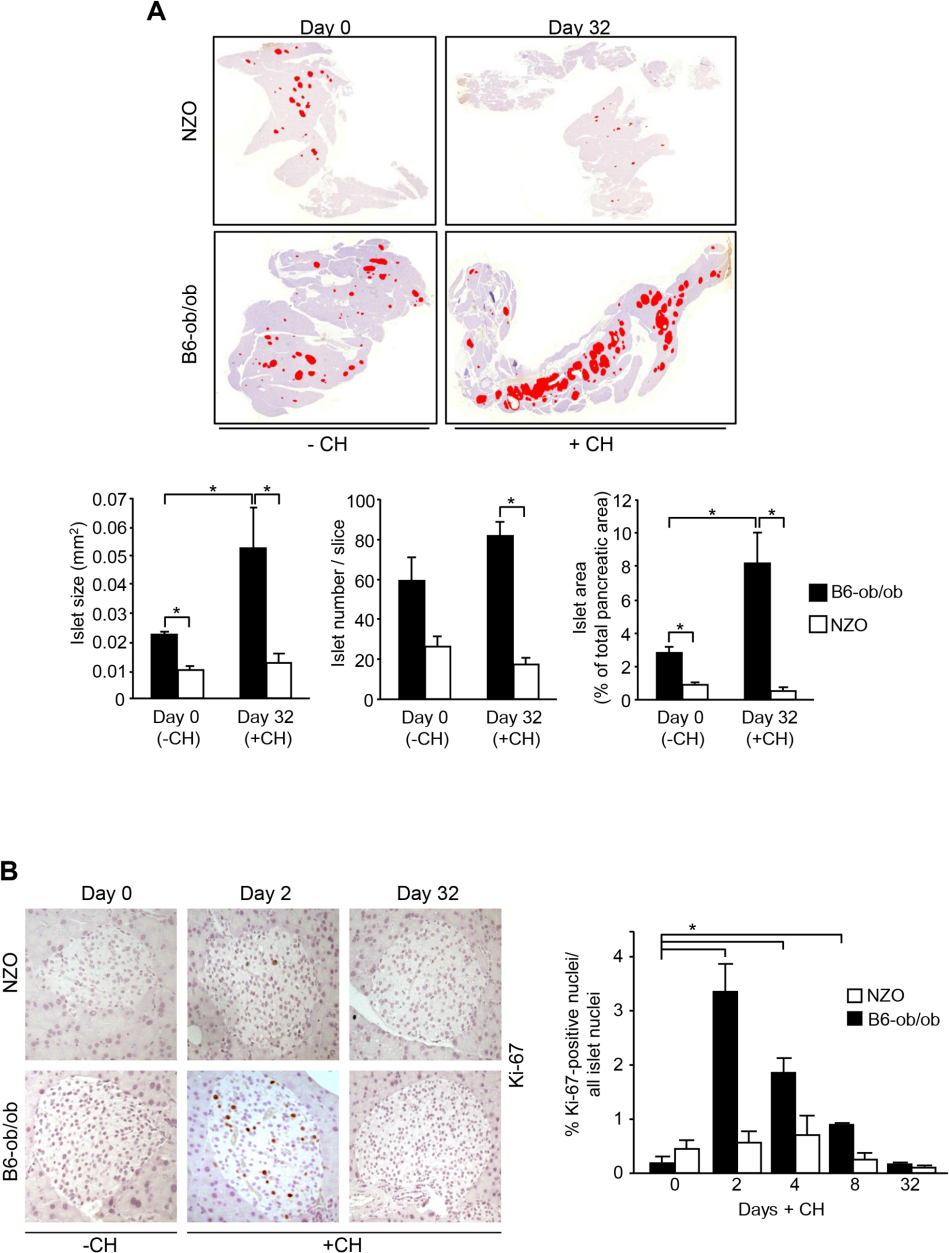
Diabetes resistance of B6-ob/ob mice is conferred by an adaptive islet hyperplasia. (A) Representative pictures of total pancreas slices from NZO and B6-ob/ob mice stained for insulin (digital red-colouring) before carbohydrate feeding (-CH) at day 0 and after 32 days of carbohydrate (+CH) feeding (upper panel). Morphometric analysis of insulin staining from 3–5 animals per time point performed in 3 cutting planes with regard to islet size (left panel), islet number per slice (middle panel), and islet area as percentage of total pancreatic area (right panel). Data are mean ± s.e.m.; *P≤0.05 (lower panel). (B) Representative immunohistochemical staining of Ki-67 in pancreatic slices from B6-ob/ob and NZO mice at the indicated time points of carbohydrate-free (-CH) or carbohydrate (+CH) feeding (upper panel). Quantification of Ki-67 positive islet cells from B6-ob/ob and NZO mice fed-CH or +CH at the indicated time points. Data represent mean ± s.e.m. of 4–6 animals, calculated from mean values of 3 cutting planes per pancreas; *P<0.05 (lower panel).

We have previously reported that a specific dietary regimen consisting of pretreatment with carbohydrate-free diet for 15 weeks and a subsequent intervention with a carbohydrate-containing diet results in completely different reactions in diabetes-susceptible NZO and diabetes-resistant B6-ob/ob mice. NZO mice develop hyperglycemia (20.2 ± 2.2 mM blood glucose at day 16) and lose most of their beta-cells by day 32 (Fig 1A, upper panels), whereas B6-ob/ob mice were able to compensate after a transient increase in blood glucose concentrations showing 8.0 ± 0.1 mM blood glucose at day 16 [12]. This might be due to a massive islet hyperplasia that was detected at the end of the intervention state as demonstrated in a representative overview of total pancreas sections of mice before and after receiving carbohydrates (Fig 1A, upper panels). Morphometric analysis demonstrated that size of islets in B6-ob/ob mice increased in response to the carbohydrate challenge rather than their number (Fig 1A, lower panels).

In order to confirm that the carbohydrate intervention induces proliferation of islet cells of B6-ob/ob mice we determined Ki-67 positive nuclei in pancreatic sections of NZO and B6-ob/ob mice that were treated with carbohydrates for 2, 4, 8, and 32 days. At no time point an induction of proliferation occurred in NZO islets, whereas B6-ob/ob islets showed a transient induction of proliferation. Highest numbers of Ki-67 positive islet cells were detected at day 2, and these numbers dropped to the initial level up to day 32 of treatment (Fig 1B).

### Differentially expressed genes in islets of diabetes-prone NZO and diabetes-resistant B6-ob/ob mice

**Fig 2:**
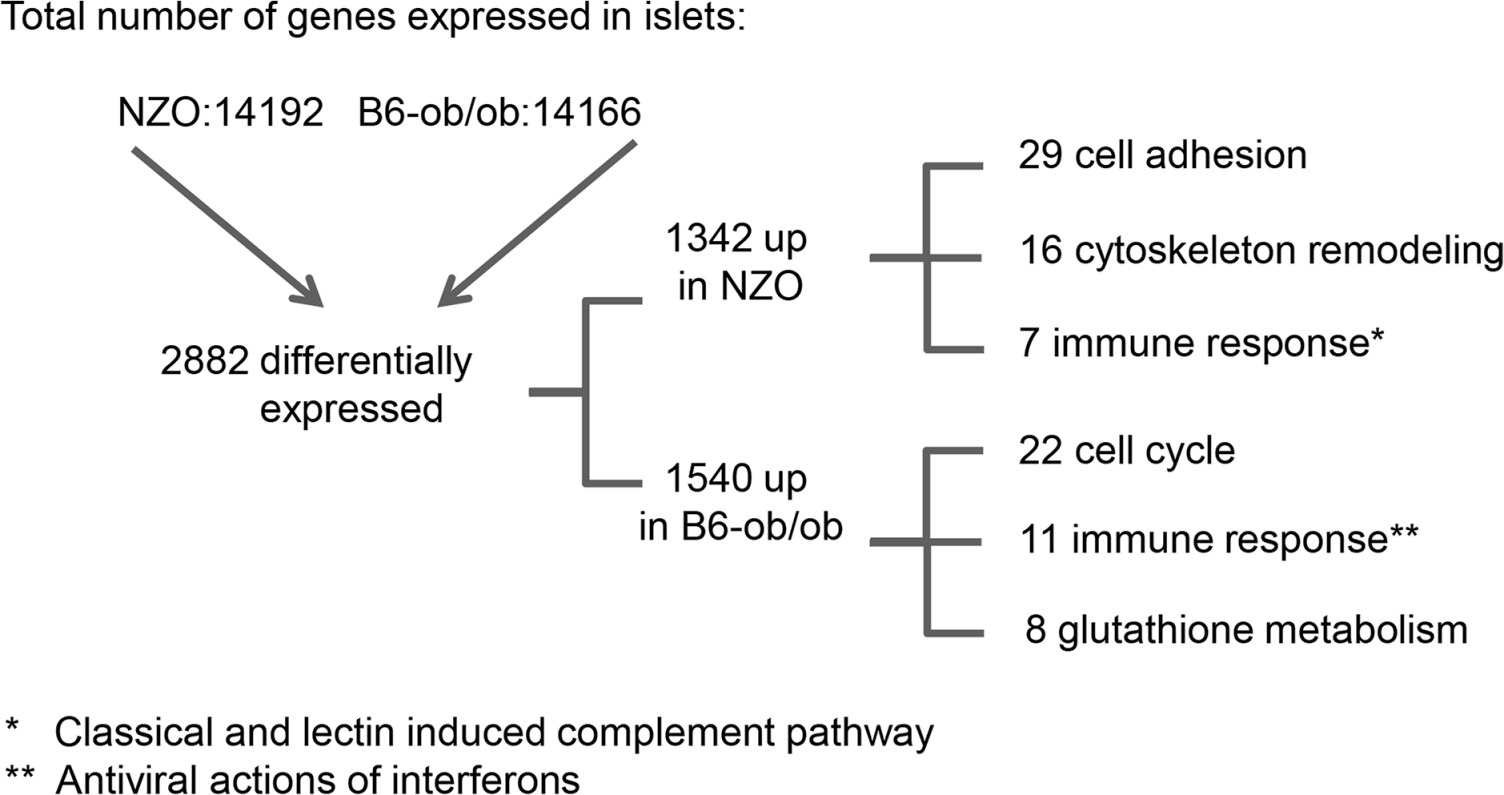
Beta-cell failure in NZO mice is associated with a modulation of cell adhesion whereas B6-ob/ob mice are protected against diabetes due to an induction of beta-cell cycle. Overview of results obtained by RNAseq-based transcriptome analysis. Transcriptome of islets from B6-ob/ob and NZO mice that were fed with a carbohydrate-free diet for approximately 15 weeks and subsequently with a carbohydrate-containing diet for 2 days was studied by RNAseq. Indicated are number of genes that are expressed in the islets, those that are differentially expressed (*P*<0.05, log2 FC > │0.6│) and the quantity of transcripts that are enriched in the indicated pathway as detected by MetaCore.

For the identification of genes and pathways that participate in the prevention of diabetes we performed RNAseq based transcriptome analyses of isolated islets of NZO and B6-ob/ob mice two days after the diet switch. MetaCore enrichment pathway analysis with 2882 differentially expressed islet genes in NZO and B6-ob/ob (log2 FC > │0.6│; *P*<0.05) revealed an upregulation of 22 genes involved in regulation of cell cycle and 8 genes of glutathione metabolism in B6-ob/ob islets, whereas NZO islets exhibited an elevated expression of 29 transcripts mediating cell adhesion and of 7 genes associated with inflammation (Table 1 and Fig 2).

**Table 1:** Main pathways up-regulated by carbohydrate feeding in B6-ob/ob and NZO mice as revealed by MetaCore enrichment pathway analysis. Shown are clusters of the main pathways upregulated in either B6-ob/ob (upper table) or NZO (lower table) islets in combination with the respective genes to be affected and their false discovery rate (FDR).

B6-ob/ob				
**Position**	**Cluster**	**Included pathways**	**Affected genes**	**FDR**
1	Cell Cycle	Regulation of G1 / S transition	*Cdkn2b*, *Ppp2r2c*, *Ccna2*, *Btrc*, *Brca1*, *Cdkn1a*, *Cdkn2a*	3.812E-02
		Chromosome condensation in prometaphase	*Ccna2*, *Ncapd2*, *Top2a*, *Ncapg2*, *Hist1h1c*, *Hist1a1e*	3.812E-02
		ATM / ATR regulation of G2 / M checkpoint	*Ccna2*, *Brca1*, *Cdkn1a*, *Clspn*, *Pkmyt1*, *Gadd45b*	5.655E-02
		Metaphase checkpoint	*Spc25*, *Kntc1*, *Bub1*, *Bub1b*, *Cenpf*, *Cenph*, *Casc5*	5.655E-02
2		Immune response: antiviral actions of Interferons	*H2-K1*, *H2-D1*, *H2-Ab1*, *H2-Eb1*, *H2-Aa*, *Oas1a*, *Oas2 Oas3*, *Mx2*, *Oas1b*, *Stat1*	3.827E-02
3		Glutathione metabolism	*Gstm1*, *Gstm2*, *Gstm5*, *Gsto1*, *Gsto2*, *Gstk1*, *Gpx2*, *Gpx3*	1.789E-01
**NZO**				
**Position**	**Cluster**	**Included pathways**	**Affected genes**	**FDR**
1	Cell adhesion	ECM remodeling	*Mmp12*, *Mmp7*, *Hbegf*, *Igf1r*, *Vtn*, *Erbb4*, *Plat*, *Fn1*, *Nid1*, *Ezr*, *Lama4*, *Lama1*, *Col3a1*, *Col1a1*	7.908E-04
		Integrin-mediated cell adhesion and migration	*Bcar1*, *Actn2*, *Pak1*, *Fn1*, *Myh14*, *Myh11*, *Mylk*, *Myl9*, *Lama1*, *Col1a1*	5.646E-02
		Cell-matrix glycoconjugates	*Lyve*, *Hapln1*, *Tnc*, *Itih1*, *Eln*, *Ambp*, *Cspg4*, *Lama1*	5.776E-02
2		Cytoskeleton remodeling TGF, WNT and cytoskeletal remodeling	*Bcar1*, *Mmp7*, *Vtn*, *Actn2*, *Smad3*, *Pak1*, *Plat*, *Fn1*, *Mylk*, *Acta2*, *Acta1*, *Actc1*, *Lama1*, *Myl9*, *Fzd1*, *Jun*	2.967E-02
3	Immune response	Classical complement pathway	*C2*, *C4a*, *C4b*, *Crp*, *Cd59b*, *Cd93*, *Trpm2*	1.807E-03
		Lectin induced complement pathway	*C2*, *C4a*, *C4b*, *Cd59b*, *Cd93*	4.842E-03

### Projection of differentially expressed islet genes to the QTL *Nob3*

**Fig 3:**
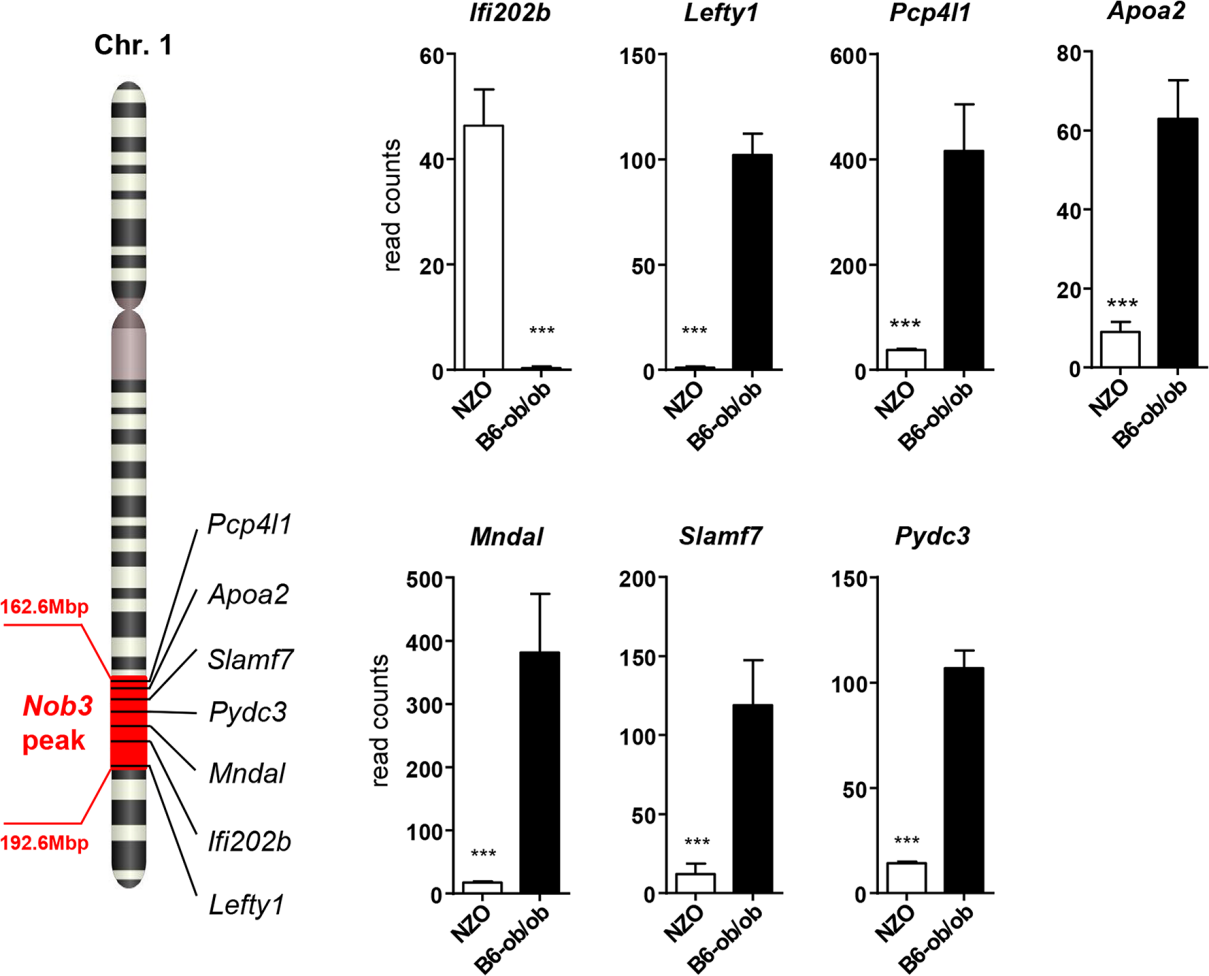
Differentially expressed islet genes in B6-ob/ob and NZO located in a QTL for type 2 diabetes. Expression levels of the indicated 7 candidates localized within the diabesity-QTL *Nob3* on Chr. 1 in islets of NZO and B6-ob/ob mice as detected by RNAseq. Data represent mean ± s.e.m. of 3 animals. Statistics were calculated by edgeR and DEseq; ***P<0.001.

In order to identify the genes responsible for the diabetes resistance of B6-ob/ob and the diabetes sensitivity of NZO mice we projected the differentially expressed islet genes to the major diabesity QTL we have recently identified in a F2 generation of NZO x C57BL/6. This QTL (*Nob3*) with LODscore values of 16.1, 16.0, 4.0 for body weight, body fat, and blood glucose, respectively was located on the distal part of Chr. 1 [13]. By defining a minimal log2 FC of │1.5│ we found 15 genes to be differentially expressed within the peak region (162.6 to 192.6 Mbp) of *Nob3* (Table 2). Among these we found 6 genes with an exclusive expression (<15% rest mRNA) in B6-ob/ob islets (*Lefty1*, *Pcp4l1*, *Apoa2*, *Mndal*, *Slamf7*, *Pydc3*) and one, *Ifi202b* solely in NZO islets (Fig 3). These fundamental differences were confirmed by qRT-PCR in a new batch of isolated islets (S1 Fig). In contrast to the RNAseq results we detected higher *Apoa2* mRNA levels in NZO islets, however, the *Apoa2* expression in B6-ob/ob islets was strikingly higher.

**Table 2:** Differentially expressed islet genes in B6-ob/ob and NZO located in a QTL (*Nob3*) for type 2 diabetes related traits that were detected by linkage studies in (NZOxB6) F2. Shown are the chromosomal localization, the log2 FC, the P-value and the accession numbers as RefSeq ID. Genes in bold are exclusively expressed either in NZO or B6-ob/ob islets (<15% rest mRNA).

Gene Symbol	Chrom	log2 FC	P-value	Refseq
***Ifi202b***	chr01	-7.50	1.58E-14	NM_008327
*Tnn*	chr01	-2.42	2.41E-06	NM_177839
*Crp*	chr01	-2.38	1.50E-05	NM_007768
*Fcer1g*	chr01	-1.61	3.01E-08	NM_010185
*Ifi203*	chr01	1.65	2.55E-12	NM_008328
*Exo1*	chr01	1.65	1.30E-05	NM_012012
*Tlr5*	chr01	1.92	7.69E-05	NM_016928
*Pyhin1*	chr01	2.06	5.42E-08	NM_175026
*Fcgr4*	chr01	2.07	3.37E-05	NM_144559
***Apoa2***	chr01	2.61	4.24E-08	NM_013474
***Pydc3***	chr01	2.70	1.37E-10	NM_001162938
***Slamf7***	chr01	2.97	1.38E-13	NM_144539
***Pcp4l1***	chr01	3.33	1.81E-30	NM_025557
***Mndal***	chr01	4.21	1.85E-38	NM_001170853
***Lefty1***	chr01	6.36	8.35E-25	NM_010094

### *Lefty1*, *Pcp4l1*, *Apoa2*, and *Ifi202b* modulate proliferation of islet cells

**Fig 4:**
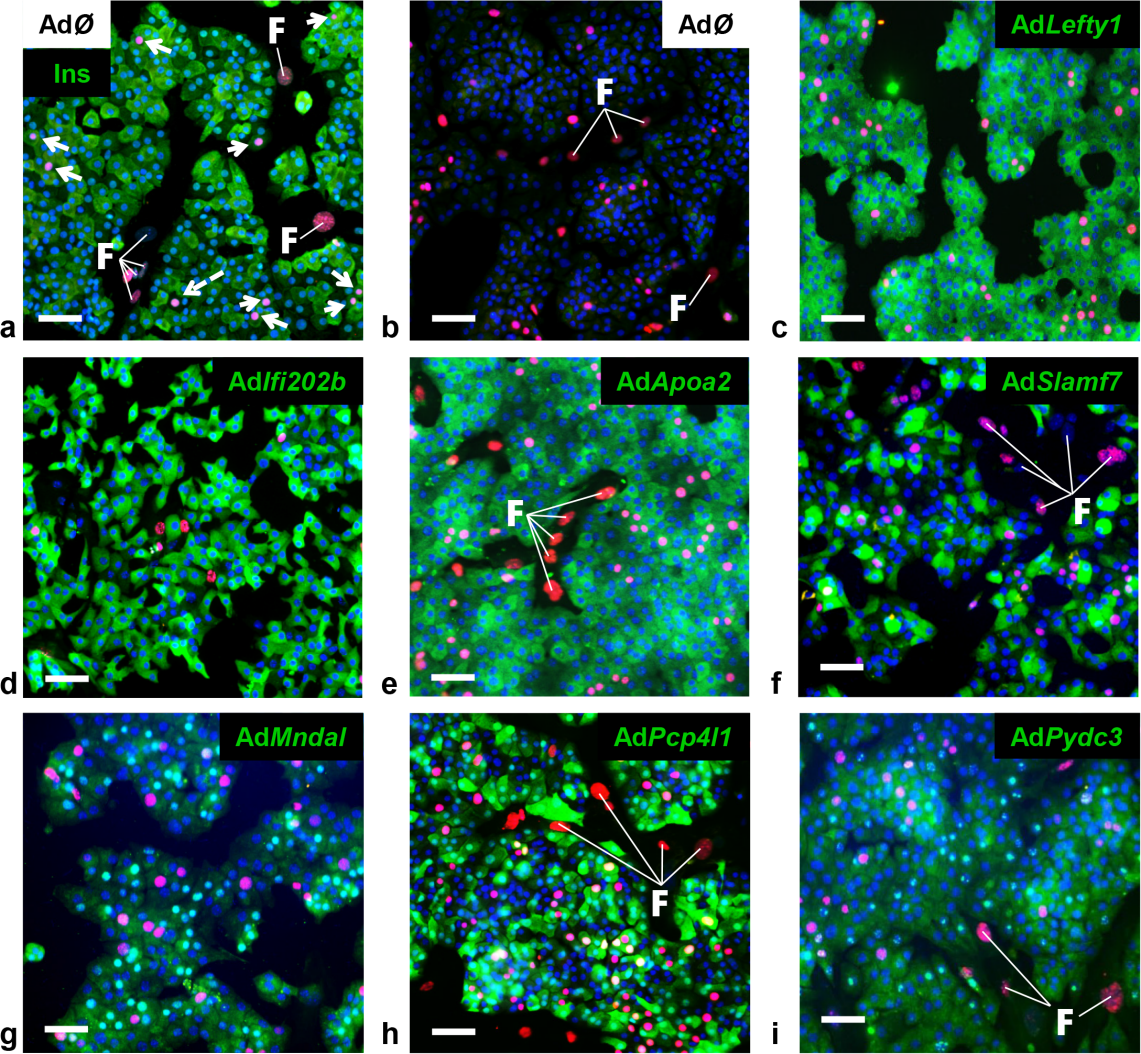
Detection of BrdU positive primary islet cells overexpressing candidate genes. Representative immunocytochemical stainings of dispersed B6 islet cells infected with empty virus (AdØ) as control or adenoviruses expressing the designated genes followed by a 72 h BrdU labeling period. (a) Co-staining of insulin (Ins; green) and BrdU (magenta). White arrows indicate BrdU-positive beta-cells; white dashed arrow indicates BrdU-positive non-beta-cells. (b-i) Co-staining of the myc-tag of indicated proteins (b: control; c: *Lefty1*, d: *Ifi202b*, e: *ApoA2*; f: *Slamf7* g: *Mndal*; h: *Pcp4l1*; i: *Pydc3*; green) and BrdU (magenta). The infection rate was between 40 and 90%. Large nuclei are fibroblast-like cells that were left out in any morphometry (white F). Blue: DNA / nuclei. Scale bar: 50 μm.

A major difference in the response to the carbohydrate challenge between NZO and B6-ob/ob islets was the lack and induction of proliferation, respectively (Figs 1 and 2). We therefore tested if genes within *Nob3* which exhibited the major expression difference in islets of both mice, *Ifi202b*, *Lefty1*, *Pcp4l1*, *Apoa2*, *Mndal*, *Slamf7*, and *Pydc3* directly modify islet cell proliferation. We overexpressed each gene in primary islet cells of B6 mice by adenoviral-mediated infection and subsequently analyzed the proliferation capacity by detecting the BrdU incorporation. In order to estimate the levels of beta-cells within primary islet cells and to test if beta-cells are able to proliferate we infected cells with an empty virus, incubated the cells with BrdU and performed a co-staining of BrdU and insulin 72 h later (Fig 4A). As expected, most of the cells were beta-cells and could be distinguished from fibroblasts due to their different shape.

**Fig 5:**
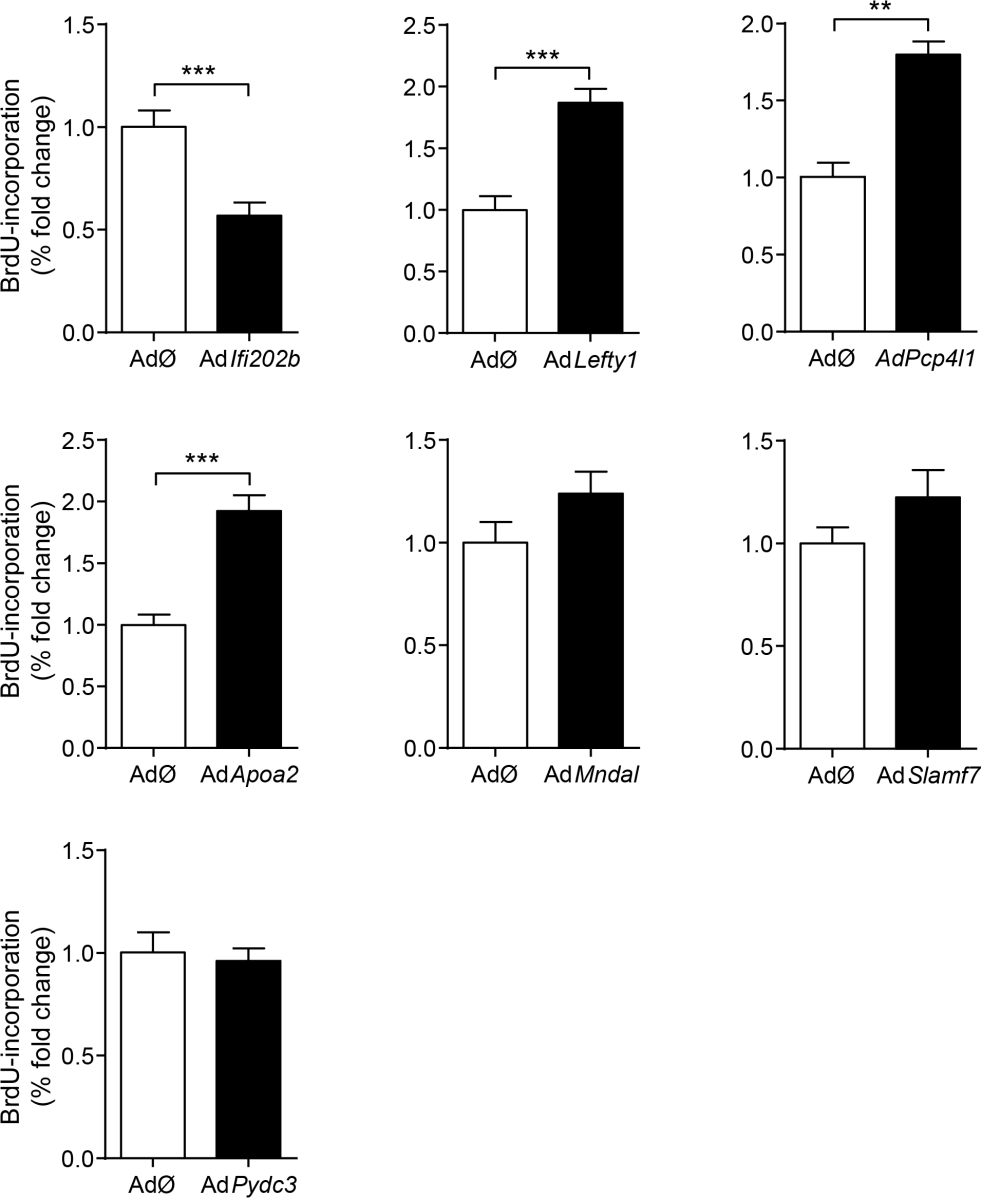
Effects of *Ifi202*, *Lefty1*, *Pcp4l1*, *Apoa2*, *Mndal*, *Slamf7*, and *Pydc3* on beta-cell proliferation. Effects of overexpression of indicated genes on BrdU incorporation in primary islet cells of B6 mice. Islets of B6 mice were isolated, dispersed and infected with adenoviruses encoding for the indicated genes. After infection, BrdU was applied for 72 h. For each candidate between 37,000 and 92,000 myc-expressing cells were evaluated. Due to variations in absolute primary islet cell proliferation depending on cell number and proper digestion, data are presented as fold change to control. Data are mean ± s.e.m. of 3–5 independent experiments **P<0.01; ***P<0.001.

We next infected primary islet cells with viruses encoding for *ApoA2*, *Lefty1*, *Pcp4l1*, *Mndal*, *Slamf7*, *Ifi202b*, and *Pydc3* in combination with BrdU incorporation. The overexpression of each candidate was visualized by immunostaining of the respective myc-tag; the infection rate was between 40 and 90% (Fig 4B–4I). Notably, every islet cell culture was accompanied by a growth of fibroblast-like cells, however, as these cells started to grow after virus treatment they were not infected and thereby not myc-positive (Fig 4). Presumably, according to individual differences of islet isolation, digestion, and growth the BrdU incorporation rate varied between the experiments in a range from 1.2 to 7.4% (mean: 3.6 ± 0.6%). In most cases BrdU was detected in insulin-positive cells [95% (AdØ), 96% (Ad*Apoa2*), 93% (Ad*Lefty1*), 91% (Ad*Pcp4l1*), 88% (Ad*Mndal*), 93% (Ad*Slamf7*), 91% (Ad*Ifi202b*), and 94% (Ad*Pydc3*)]. Infection of cells with the *Lefty1*, *Pcp4l1*, and *Apoa2* encoding viruses increased BrdU incorporation by 87%, 80%, and 92%, respectively; overexpression of *Ifi202b* decreased the number of BrdU positive cells by 43%. All other candidates were without effect (Fig 5).

### Genetic association of human *LEFTY1* with altered insulin release

Previous human studies have linked type 2 diabetes traits to chr. 1q21-24 in multiple samples [14]. It was shown that seven variants increased the diabetes risk significantly through 5 pairs of interactions. One significant variant pair was a SNP in *APOA2* (rs6413453) interacting with calsequestrin-1 (CASQ1; rs617698) [14]. As no association was described for *LEFTY1* so far we tested a population of 1,865 individuals with a median age of ~39 years and a BMI of 28.5 kg/m² that was recruited from the Tübingen Family (TUEF) study for type 2 diabetes and characterized before [15]. The participants were genotyped for 10 SNPs tagging the *LEFTY1* gene and 2 kb of its 5’-flanking region for association analysis. After adjusting for gender, age, and oGTT-derived insulin sensitivity the minor T-allele of rs3806259 was nominally associated with elevated insulin levels during oral glucose tolerance tests (*P* = 0.0034), whereas the minor allele of rs360060 was nominally associated with a lower insulin release (*P* = 0.0052; S2 Fig). Both SNPs were also nominally associated with elevated (*P* = 0.0593) and reduced (*P* = 0.0111) C-peptide release, respectively.

## Discussion

The present data demonstrated that islets of diabetes-resistant and diabetes-prone mouse strains respond inversely to a glucose challenge: In B6-ob/ob islets, the induction of proliferation resulted in a strong increase in beta-cell mass, which appears to be responsible for an increase in plasma insulin concentration and a sufficient control of blood glucose even under glucotoxic conditions, whereas NZO islets lack the ability to initiate cell cycle progression and to maintain the survival pathway. We identified 7 genes within the diabesity QTL *Nob3* which exhibit a nearly exclusive expression in islets of either diabetes-susceptible or diabetes-resistant mice. Three genes, *Lefty1*, *Pcp4l1*, and *Apoa2* are the most likely candidates to suppress and one gene, *Ifi202b* to accelerate T2D and beta-cell failure in mice.

To clarify the genetic basis of the inverse reaction of NZO and B6-ob/ob islets on the glucose challenge and to identify diabetes suppressor and enhancer genes we aligned the transcriptome data to the major susceptibility locus identified in former association studies from a NZOxC57BL/6J cross, the *Nob3* with a LOD score of 16 for adiposity and of 4 for hyperglycemia [13]. In total, 15 differentially expressed islet genes are located in the *Nob3*. Focusing on transcripts with the strongest effect revealed six genes (*ApoA2*, *Lefty1*, *Mndal*, *Pcp4l1*, *Pycd3*, and *Slamf7*) to be exclusively expressed in B6-ob/ob and one, *Ifi202* in NZO islets. The most prominent molecular alterations were identified for *Ifi202b* and *Lefty1*. *Ifi202b* which was recently identified as an obesity gene in our group by positional cloning carries a microdeletion that includes the promoter and the first exon of *Ifi202b* and results in a loss of *Ifi202b* expression in most tissues from B6 mice such as white adipose tissue, liver, and skeletal muscle [5], and as shown here, also in islets. Since the overexpression of *Ifi202b* in primary islet cells suppressed proliferation we conclude that it participates in the inability of NZO mice to compensate with an increased beta-cell mass when blood glucose levels rise. Consistent with this conclusion, Clarke et al. (2010) demonstrated that the overexpression of *IFI16*, a human orthologue of *Ifi202b*, decreases cellular proliferation by growth arrest in the G1 phase of the cell cycle [16].

**Fig 6:**
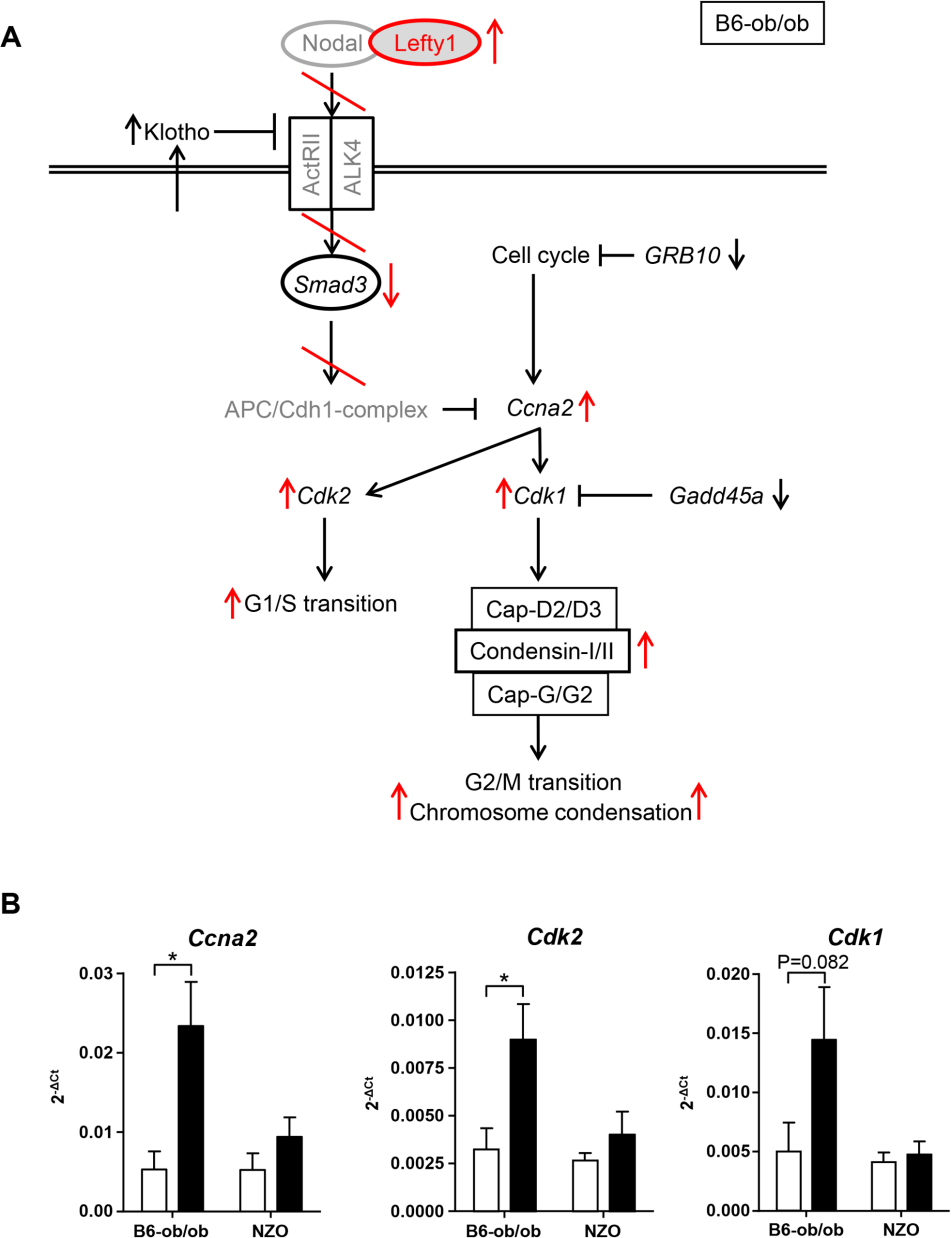
Schematic representation of pathways that are affected in B6-ob/ob and NZO islets. (A) Based on pathway analysis performed with differentially expressed genes detected by RNAseq of B6-ob/ob and NZO islets the Lefty/Nodal/Smad3 pathway appears to play a central role for the initiation of beta-cell proliferation in B6-ob/ob. Red arrows and lines indicate the regulation of signaling molecules in B6-ob/ob beta-cells in response to Lefty1. The presence of Lefty1 inhibits Nodal signaling via the ActRII/ALK4 receptor leading to a reduced activity of the anti-proliferative protein Smad3 and ultimately to an induction of proliferation via Cyclin A2 (Ccna2), Cdk2 and Cdk1. Proteins in grey indicate candidates that were not significantly different in the RNAseq analysis. Black arrows indicate the regulation (RNAseq) of additional molecules that could be involved in the pathway. (B) Expression of the indicated cell-cycle transcripts in islets of NZO and B6-ob/ob mice before and after feeding carbohydrates for 2 days as detected by qRT-PCR. Data are mean ± s.e.m. of 4–6 animals; *P≤0.05.

Lefty1, one candidate exclusively expressed in islets of diabetes-resistant B6-ob/ob mice, is an atypical member of the TGF-β family that inhibits Nodal by direct binding to Nodal [17–19]. Recently, Zhang et al. (2008) reported that Nodal and Lefty1 are expressed in the pancreas during embryogenesis and islet regeneration and demonstrated that Lefty1 activates MAPK and AKT in vitro [20]. Our *in vitro* studies in primary islet cells clearly supported that *Lefty1* expression increases proliferation as detected by an increased BrdU incorporation. As Lefty1 is a central regulator of the Smad3 pathway by antagonizing Nodal, we screened our RNAseq and pathway enrichment data for downstream transcripts and indeed found several targets exhibiting an altered expression as indicated in Fig 6A. Nodal activates Smad3, a transcription factor which exhibits a higher expression in NZO than in B6-ob/ob islets (Figs 6A and S3). Smad3 is a tumor suppressor which inhibits cell proliferation and promotes apoptosis [21]. Accordingly, the lower expression of *Smad3* in B6-ob/ob islets as a consequence of the presence of Lefty1 could trigger beta-cell proliferation in this diabetes-resistant model (Fig 6A). Specifically, in pancreatic beta-cells Smad3 activation was shown to promote the Cdc25a ubiquitination and degradation via two ubiquitin ligases, the SCFbeta-TrCP-complex and the anaphase-promoting complex (APCCdh1) [22]. Cyclin A2 (*Ccna2*), another target to be suppressed by the APCCdh1-complex [23,24], is elevated specifically in B6-ob/ob islets in response to carbohydrate feeding and plays a pivotal role in cell-cycle regulation together with the targets *Cdk1* and *Cdk2* that are also upregulated as determined by qRT-PCR (Fig 6B) [25]. In contrast, without *Lefty1* NZO islets display higher *Smad3* levels that might participate in raising the expression of ECM molecules such as collagen and fibronectin [26,27] and an increased expression of *Mmp7* and *Mmp12* that cleave the ECM proteins vitronectin and laminin (Fig 2 and Table 1).

We found that two SNPs in human *LEFTY1* were nominally associated with altered insulin release in a glucose tolerance test, but not with glycaemia or diabetes. This association seems to suggest that *LEFTY1* could be a minor contributor to the pathogenesis of the human disease. However, the finding needs validation in an independent study before a definitive conclusion can be drawn. No significant association between diabetes-related traits and the genotype of 15 SNPs in *IFI16*, the human orthologue of *Ifi202b*, was found.

Besides *Lefty1*, *Apoa2* showed an excessive expression in the B6-ob/ob islet and in primary islet cells also a positive effect on proliferation. In fact SNPs in human *APOA2* were shown to associate with type 2 diabetes in a Utah case-control sample [14]. However, this finding was not confirmed in French Caucasian subjects [28]. *Pcp4l1* (Purkinje cell protein 4 like 1) was also sufficient to increase BrdU incorporation in primary islet cells. Little is known about the function of *Pcp4l1*. It is described to be expressed during early brain development where it is localized to the midbrain-hindbrain boundary in a pattern that partially overlaps with *Wnt1*, *Pax2* and *Fgf8* expression domains [29]. Its closest related protein is Pcp4/PEP-19, a calmodulin-binding protein exhibiting a neuronal IQ motif. However, in contrast to Pcp4/PEP-19, Pcp4l1 lacks the capacity of calmodulin binding presumably due to a nine-residue glutamic acid-rich sequence that is located in proximity to the IQ motif [30]. A link of *Pcp4l1* and diabetes has not been described so far.

In summary, our data indicate that *Lefty1*, *Apoa2*, *Pcp4l1* and *Ifi202b* modify beta-cell proliferation, and suggest that the alteration of their expression contributes to the development of severe diabetes in the NZO mouse strain.

## Material and Methods

### Experimental animals

Male NZO/HIBomDife mice (R. Kluge, German Institute of Human Nutrition, Nuthetal, Germany) from our own colony and male B6.V-*Lep* ^*ob/ob*^/JBomTac (ob/ob) mice (Charles River Laboratories, Italy) were housed in groups of at most five per cage (type II/III macrolon) at a temperature of 21 ± 2°C with a 12:12 h light-dark cycle (lights on at 6:00 a.m.). Animals had free access to food and water at any time.

### Diets and study design

Starting at the age of 5 weeks onwards, all mice received a carbohydrate-free diet (-CH) containing 68% fat (w/w) and 20% protein (w/w) with a total metabolisable energy of 29.3 kJ/g. At the age of 18 ± 1 weeks, subgroups of the animals received a carbohydrate-enriched diet (+CH) containing 28% fat (w/w), 20% protein (w/w) and 40% (w/w) metabolisable carbohydrates for either 48 hours or 32 days. The metabolisable energy content of the +CH was 21.9 kJ/g. Due to the soft texture of the diets mice had access to wooden gnawing sticks in order to avoid excessive teeth growth. The ingredients of the diets are given in S1 Table.

### Immunohistochemistry of pancreatic islets

Pancreatic tissue excised immediately after Isofluran anesthesia, was fixed in 4% formaldehyde for 24 h and embedded in paraffin according to standard procedures. Longitudinal serial sections (2–3 μm thickness, sampling intervals 100 μm) were prepared. After re-hydration, slices were incubated with primary antibodies against insulin (1:50,000; 1 h; room temperature; Sigma) and Ki-67 (1:50; overnight; 4°C; Dako; Hamburg; Germany). Bound primary antibodies were detected with Histofine Immuno-POD Polymer (Nichirei Biosciences, Tokyo, Japan) against mouse or rat, followed by Diaminobenzidine (DAB) incubation for visualization of protein localization. Detection of apoptotic cells by a TUNEL-staining was performed by the ApopTag Plus Peroxidase *In Situ* Apoptosis Detection Kit (Billerica, US) according to manufacturer’s instructions.

Microscopic investigation and photo documentation were performed with the combined light and fluorescence microscope ECLIPSE E-100 (Nikon, Düsseldorf, Germany) in combination with video camera equipment (CCD-1300CB, Vosskühler, Osnabrück, Germany) and the analysis system NIS elements (Nikon). Total pancreas photographs were made by a MIRAX MIDI scanner (Zeiss, Oberkochen, Germany) and evaluated by a software package (AxioVision, Zeiss).

### Quantitative analysis of islet stainings

Morphometric analyses of Ki-67 staining were performed by counting the number of positive nuclei in 3 sectional planes of one pancreas with the help of the analysis software NIS elements (Nikon). Mean values derived from each individual mouse were used to calculate mean ± s.e.m. (4–6 animals in each group). Insulin stainings were used to determine islet size, islet number and islet mass of B6-ob/ob and NZO mice. Similarly, quantifications were made from 3 sectional planes of one pancreas and means from each animal were used to calculate mean ± s.e.m. (3–5 animals per group). This analysis was realized by using the AxioVision software package (Zeiss). All morphometric quantifications were conducted via an unbiased stereological approach.

### Islet isolation and transcriptome analysis

Isolation of islets was performed by a modified protocol of Gotoh et al. (1990) from 18 ± 1 week old NZO and B6-ob/ob mice [31]. Briefly, the pancreas was perfused by injection of 3 ml of Collagenase-P (Roche, Mannheim, Germany) (1 mg/mL) in Hank’s buffered salt solution (HBSS) also containing 25 mM HEPES and 0.5% (w/v) BSA into the common bile duct. Subsequently, the perfused pancreas was digested in 2 ml of collagenase solution for 8 to 10 min at 37°C. With the help of a cannula (18G x 11’2) islets were mechanically detached from exocrine parts, and washed for several times with HBSS and RPMI 1640 containing 10% FCS and 100 U/mL penicillin/streptomycin (PeSt). Islets were passaged for several times in RPMI 1640 by hand-picking and finally collected in Eppendorf tubes. Total islet RNA preparation was performed with the RNAqueous-Micro Kit (Life Technologies, Darmstadt, Germany). In order to apply high-quality RNA for RNAseq analysis we assessed RNA integrity by using a Bioanalyzer and an appropriate kit (RNA6000 nano kit, Agilent, Santa Clara, USA). All preparations were made according to manufacturer’s recommendations. Only RNA with a minimal RNA integrity number of 8.0 (RIN) was used. RNA concentration was measured in 1 μL with a spectrophotometer (NanoDrop ND-100 UV/Vis, PEQLAB, Erlangen, Germany) at 260 nm. RNA sequencing and evaluation of data was performed by LGC genomics (Berlin, Germany).

### Quantitative real-time PCR

Total islet-RNA of NZO and B6-ob/ob mice fed with the carbohydrate-free diet up to the age of 18 weeks and with or without the carbohydrate-containing diet for 2 days was isolated with the RNAqueous-Micro Kit (Life Technologies, Darmstadt, Germany). Subsequently, first-strand cDNA synthesis was performed with the whole amount of RNA, random hexamer primer and GoScript reverse transcriptase (Invitrogen). qRT-PCR was performed with 12.5 ng cDNA in an Applied Biosystems 7500 Fast Real-time PCR system with TaqMan Gene Expression Assays (Life Technologies, Darmstadt, Germany) and the TaqMan Gene Expression Master Mix. Data were normalized to the expression of cyclophilin (*Ppia)* as endogenous control (ΔC_t_-Method) [32].

### Overexpression of candidates in primary islet cells and BrdU assay

For overexpression of candidates in primary islet cells islets from C57BL/6 mice fed a high-fat diet until an age of 20 ± 2 weeks were isolated and recovered for 48 hours in RPMI 1640 (10% FCS, 1% PeSt) in humidified 5% CO_2_, 95% air at 37°C. Islets were digested with Accutase (GE Healthcare, Buckinghamshire, GB) in a volume of 75 μL per 100 islets for 6 min at 37°C and cells separated by aspirating. The digestion was stopped by addition of RPMI 1640 (10% FCS; 1% PeSt), cells were centrifuged (1000xg, 5 min, RT) and the supernatant removed. Resuspended cells were seeded on poly-L-lysine coated coverslips in 24 well plates at a corresponding amount of 1.5 x 10^5^ cells/well. Cells were maintained in RPMI 1640 (10% FCS, 1 & PeSt) containing 11 mmol/L glucose for 3–4 days. Infection was carried out by a 24 h incubation with adenoviruses carrying cDNA from *Pydc3* (MOI100), *Lefty1* (MOI100), *Slamf7* (MOI10), *Mndal* (MOI100), *Apoa2* (MOI100), *Pcp4l1* (MOI100) (Vector Biolabs) and *Ifi202b* (MOI500) (ViraQuest, North Liberty, USA) under the control of a CMV-promotor and in conjunction with the corresponding empty viruses. Due to the very low proliferation capacity of primary islet cells BrdU labeling (100 μmol/L) was performed for 72 h after virus infection as described by Tsukiyama and colleagues [33]. Afterwards, cells were fixed with 4% paraformaldehyde. After permeabilization of cell membranes (0.2% saponin), DNA denaturation (2 M HCl) and trypsinization (0.1% trypsin) cells were incubated with primary antibodies against BrdU (1:500, 1 h, room temperature; Sigma) plus anti myc-antibody (1:500, Santa Cruz, USA). Bound primary antibodies were detected with fluorophor-labeled secondary antibodies against rat (Alexa Fluor546, 1:400, Invitrogen) and rabbit (Alexa Fluor488, 1:400, Invitrogen) and documented with a confocal microscope (TCS SP-2-Confocal Laser scanning microscope, Leica Microsystems, Heidelberg, Germany). Statistical evaluation was performed by blinded quantification of 10–12 photographs of at least 2 coverslips per infection and mean ± s.e.m. was calculated from 4–5 independent experiments.

### Human subjects and measurement of insulin release

A study population of 1,865 individuals with complete insulin, C-peptide, and glucose measurements during a standard 5-point oral glucose tolerance test (oGTT) was recruited from the Tübingen Family study for type 2 diabetes [15]. From these data, insulin release was estimated as area under the curve (AUC) insulin_0–30_ divided by AUC glucose_0–30_ and as AUC C-peptide_0–30_ divided by AUC glucose_0–30_. AUCs were calculated according to the trapezoid method. oGTT-derived insulin sensitivity was calculated as proposed by Matsuda and DeFronzo [34].

### Single nucleotide polymorphism (SNP) selection and genotyping

Based on publicly available information from 1000 Genomes (http://browser.1000genomes.org/Homo_sapiens/Info/Index), we analysed the complete human *LEFTY1* and *IFI16* genes and 2 kb each of their 5’-flanking regions and identified 10 and 15, respectively, non-linked representative SNPs tagging all other common SNPs (minor allele frequency ≥ 0.1) in these loci with r² ≥ 0.8. The tagger function of Haploview 4.2 (http://www.broadinstitute.org. haploview/haploview) was used. The 10 *LEFTY1* tagging SNPs, i.e., rs7551003 G>T, rs72754996 G>A, rs193327 C>G, rs3806259 C>T, rs2013363 G>T, rs360074 G>A, rs10915896 C>T, rs360073 G>A, rs360060 G>A, and rs360058 G>T, were all non-coding and located in the 5’-flanking region of the gene. The 15 non-coding *IFI16* tagging SNPs were rs72704962 T>C, rs1465175 G>T, rs4657618 C>T, rs1417806 A>C, rs856077 C>A, and rs1417805 T>G in the 5’-flanking region, rs2276404 A>G, rs856064 C>T, and rs12727764 G>T in intron 1, rs2106095 T>A, rs12087333 A>G, rs74122213 T>C, and rs12061401 C>T in intron 6, and rs1633262 T>C and rs1772414 A>G in intron 7 of the gene. The tagging SNPs were genotyped in blood-derived genomic DNA using Sequenom’s massARRAY system (Sequenom, Hamburg, Germany) or TaqMan allelic discrimination assays (Applied Biosystems, Foster City, CA, USA).

### Statistics

RNAseq read counts were tested by 2 statistical evaluations (DEseq [35]), and edgeR [36] of the R package in combination with an error correction by Benjamini-Hochberg. All comparisons were considered significantly different at P<0.05 and a log2 FC >│0.6│and │1.5│, respectively. Differences in morphometric analysis, qRT-PCRs and BrdU-Assays were tested with the non-parametric Kruskal-Wallis test and corrected for multiple comparisons by Dunn’s. For human association studies skewed data were log_*e*_-transformed in order to approximate normal distribution. Multiple linear regression analysis (standard least squares method) was performed with insulin release as outcome variable, the SNP genotype (in the additive inheritance model) as independent variable, and gender, age, and insulin sensitivity as confounding variables. To account for multiple testing (25 SNPs tested in parallel), a Bonferroni-corrected P-value <0.002 was considered statistically significant. For these analyses, JMP 10.0 (SAS Institute, Cary, NC, USA) was used.

### Ethics statement

All animals were kept in accordance with the NIH guidelines for the care and use of laboratory animals. All experiments were approved by the Ethics Committee of the State Ministry of Agriculture, Nutrition and Forestry (State of Brandenburg, Germany). The approval numbers were: V3-2347-31-2011 and 2347-26-2014.

## Supporting Information

S1 Fig Verification of an exclusive expression of genes localized within the diabesity-QTL *Nob3* on Chr. 1 in islets of NZO and B6-ob/ob mice fed a carbohydrate-containing diet (+CH) for 2 days after a 15 week carbohydrate deprivation (-CH) as determined by qRT-PCR. Data represent mean ± s.e.m. of 5–6 animals per group. *P<0.05, **P<0.01, ***P<0.001.
(PDF)

S2 Fig Association of *LEFTY1* SNPs rs3806259 (C/T) and rs360060 (G/A) with insulin release in humans. Insulin release was calculated from plasma insulin and glucose concentrations during a 5-point oGTT. Data from 1,865 subjects are shown. All insulin release data were adjusted for gender, age, and oGTT-derived insulin sensitivity using multiple linear regression models. Grey shading represents the 95% confidence interval of individual data. AUC: area under the curve; Glc: glucose; Ins: insulin.
(PDF)

S3 Fig *Smad3* expression in islets of B6-ob/ob and NZO mice as detected by RNAseq. Data represent mean ± s.e.m. of 3 animals calculated by edgeR and DEseq; *P<0.05.
(PDF)

S1 Table Ingredients of the diets. (PDF)
