# Supplementary figures and images for: Identification of Four Mouse Diabetes Candidate Genes Altering β-Cell Proliferation
Source: PLoS Genet. 2015 Sep 8;11(9):e1005506. doi: 10.1371/journal.pgen.1005506 (PMC4562707; doi:10.1371/journal.pgen.1005506)

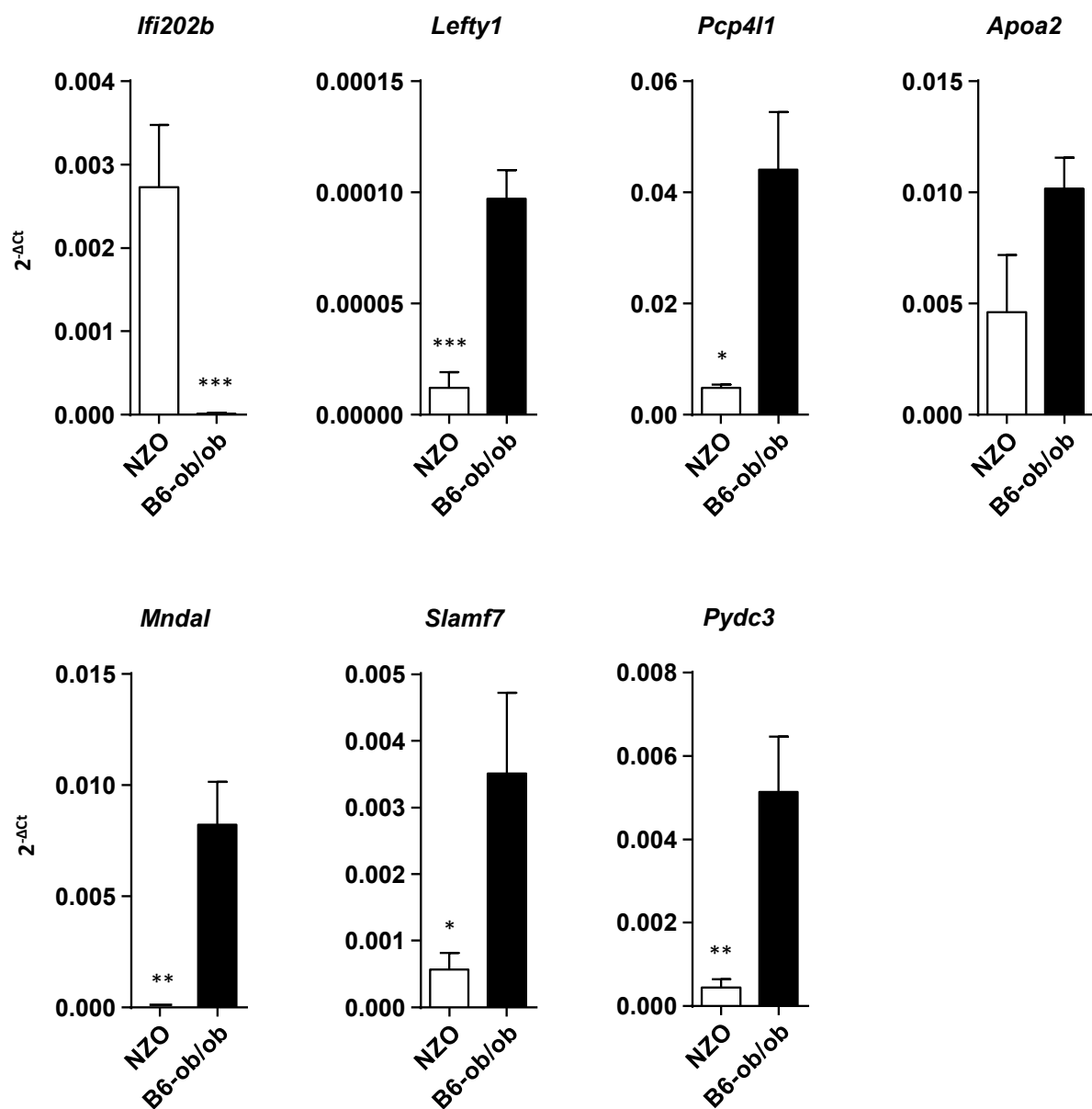

Supplement: S1 Fig — Data represent mean ± s.e.m. of 5–6 animals per group. *P<0.05, **P<0.01, ***P<0.001. (PDF) [file pgen.1005506.s001.pdf]

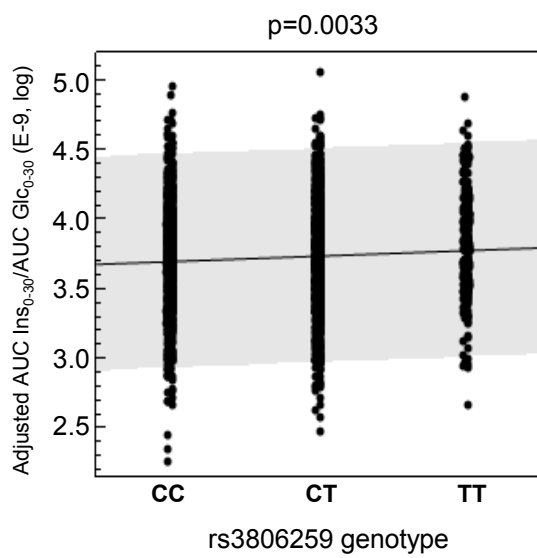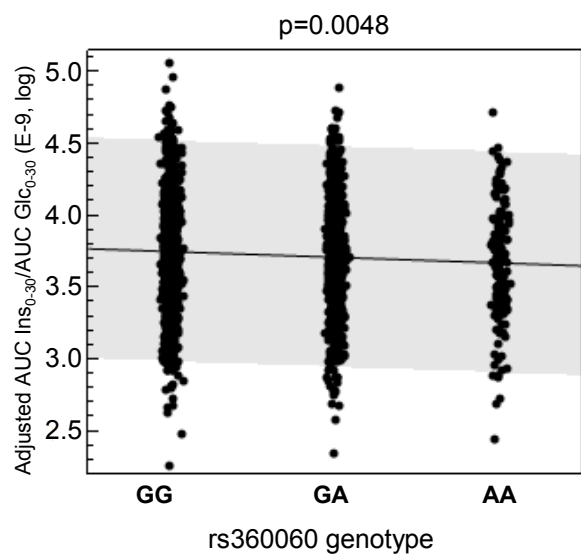

Supplement: S2 Fig — Insulin release was calculated from plasma insulin and glucose concentrations during a 5-point oGTT. Data from 1,865 subjects are shown. All insulin release data were adjusted for gender, age, and oGTT-derived insulin sensitivity using multiple linear regression models. Grey shading represents the 95% confidence interval of individual data. AUC: area under the curve; Glc: glucose; Ins: insulin. (PDF) [file pgen.1005506.s002.pdf]

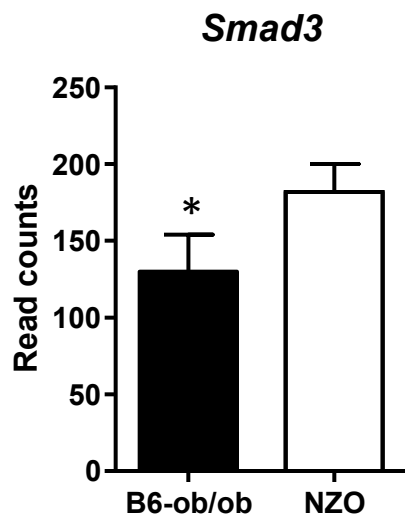

Supplement: S3 Fig — Data represent mean ± s.e.m. of 3 animals calculated by edgeR and DEseq; *P<0.05. (PDF) [file pgen.1005506.s003.pdf]
