# Supplementary material for: Identification of Four Mouse Diabetes Candidate Genes Altering β-Cell Proliferation
Source: PLoS Genet. 2015 Sep 8;11(9):e1005506. doi: 10.1371/journal.pgen.1005506 (PMC4562707; doi:10.1371/journal.pgen.1005506)
Supplement: S1 Table — (PDF) [file pgen.1005506.s004.pdf]

Table S1
Ingredients of the diets

Ingredient	Content (%, w/w)		Manufacturer
	-CH	+CH	
Casein	20	20	Bayerische Milchindustrie eG, Landshut
Palm fat	33.5	13.5	Ostthüringer Nahrungsmittelwerk, Gera
Safflower oil	0.5	0.5	Kunella Feinkost, Cottbus
Linseed oil	0.5	0.5	Kunella Feinkost, Cottbus
Microcellulose	5	5	J. Rettenmaier und Söhne, Ellwangen-Holzmühle
Mineral mixture	5	5	Altromin, Lage
Vitamin mixture	2	2	Altromin, Lage
Sucrose	0	10	Pfeifer & Langen KG, Cologne
Starch	0	30	Kröner Stärke, Ibbenbüren

Macronutrient content	- CH	+ CH
Total fat (% of total energy)	88.5	51.4
Carbohydrates (% of total energy)	0	32.4
Protein (% of total energy)	11.5	16.2
Energy density (kJ/g)	29.3	21.9
